# Liquid-liquid phase separation in DNA double-strand breaks repair

**DOI:** 10.1038/s41419-023-06267-0

**Published:** 2023-11-15

**Authors:** Yun-Long Wang, Wan-Wen Zhao, Jie Shi, Xiang-Bo Wan, Jian Zheng, Xin-Juan Fan

**Affiliations:** 1 Henan Provincial Key Laboratory of Radiation Medicine, The First Affiliated Hospital of Zhengzhou University, Zhengzhou, Henan 450052 PR China; 2 Academy of Medical Sciences, Zhengzhou University, Zhengzhou, Henan 450052 PR China; 3 Department of Radiation Oncology, The First Affiliated Hospital of Zhengzhou University, Zhengzhou, Henan 450052 PR China; 4 Department of Radiation Oncology, The Sixth Affiliated Hospital, Sun Yat-sen University, Guangzhou, Guangdong 510655 PR China; 5 GuangDong Provincial Key Laboratory of Colorectal and Pelvic Floor Diseases, The Sixth Affiliated Hospital, Sun Yat-sen University, Guangzhou, Guangdong 510655 PR China; 6 Department of Pathology, The Sixth Affiliated Hospital, Sun Yat-sen University, Guangzhou, Guangdong 510655 PR China

**Keywords:** Double-strand DNA breaks, DNA damage response

## Abstract

DNA double-strand breaks (DSBs) are the fatal type of DNA damage mostly induced by exposure genome to ionizing radiation or genotoxic chemicals. DSBs are mainly repaired by homologous recombination (HR) and nonhomologous end joining (NHEJ). To repair DSBs, a large amount of DNA repair factors was observed to be concentrated at the end of DSBs in a specific spatiotemporal manner to form a repair center. Recently, this repair center was characterized as a condensate derived from liquid-liquid phase separation (LLPS) of key DSBs repair factors. LLPS has been found to be the mechanism of membraneless organelles formation and plays key roles in a variety of biological processes. In this review, the recent advances and mechanisms of LLPS in the formation of DSBs repair-related condensates are summarized.

## Facts

- Liquid-liquid phase separation (LLPS) has emerged as a key mechanism underlying the formation of liquid-like membraneless structures in cells.
- DNA double-strand breaks (DSBs) are the biggest threat to genome integrity, which are typically repaired by HR and NHEJ.
- In this review, we summarized the roles of LLPS in DSBs repair.

## Open questions

- What are the general features of LLPS, such as the characteristics, mechanisms, regulators, and functions?
- Does LLPS participate in the process of DSBs repair?
- What are the functions of LLPS in the compartmentalization of DSBs repair centers? How are these condensates induced by DNA damage?

## Introduction

A cell can be thought of as a busy “factory”, running a variety of different tasks at the same time. To ensure the accuracy of independent tasks, a cell is compartmentalized into different departments for each specialized task. The main form of compartmentation is achieved by the membranes, which separate a cell into individual spaces by surrounding like walls. As well known, some big cellular organelles, including the nucleus, mitochondria, Golgi bodies, and endoplasmic reticulum, are compartmentalized with membranes [1]. However, there are many organelles, such as nucleolus, paraspeckle, and stress granules, which maintain stable structure and separated spaces without membranes [2], while the underlying mechanisms remain a mystery. Recently, this mystery driving force has been ascribed to the liquid-liquid phase separation (LLPS) of macromolecules.

The concept of LLPS was first raised by Dr. Anthony A. Hyman in 2009 [3]. They reported that P granules exhibited liquid-like properties, which was referred as liquid-liquid phase separation in their following studies. After ten years of study, LLPS has been reported to play key roles in not only the formation of canonical membraneless organelles, but also almost every locally performed biological process, including heterochromatin formation, transcription activation, RNA decay, signal transduction, and DNA repair [4–8].

Given the participation and activation of many nucleases, the task of DNA damage repair needs to be restricted in a compartmentalized space to ensure genome stability. As the most fatal type of DNA damage, DNA double-strand breaks (DSBs) are the main threat to genome integrity. DSBs are mainly repaired by homologous recombination (HR) and nonhomologous end joining pathway (NHEJ), involving dozens of repair factors [9–12]. The condensation of DNA damage response and repair factors at the DSBs ends has been observed over thirty years [13–15], which is considered as the result of their direct or indirect interaction with the factors already present at DSBs. However, there are still some phenomena that can’t be explained by this theory, e.g., the dynamic exchange of 53BP1 and RAD52 at DNA damage foci [16, 17]; the spatiotemporal changes of 53BP1 and BRCA1 foci [18–20]. Here, we provide a glance over recent advances in LLPS study, discuss the role of LLPS in DSBs repair factors condensation and its mechanism, and how specific LLPS are induced at DSBs. Overall, this review highlights the key functions of LLPS in the compartmentalization of DSBs repair center.

## Brief review on liquid-liquid phase separation

### Characteristics of liquid-liquid phase separation

Recently, LLPS has emerged as an underlying mechanism that regulates the formation of liquid-like membraneless structures in cells, which are also referred to as biomolecular condensates. Condensates produced by LLPS, including the nucleolus, paraspeckles, stress granules, P granules, Cajal bodies, and others, provide a compartmentalized space suitable for various biochemical reactions within a cell. When biomolecules undergo LLPS, they separate into a highly concentrated dense phase and a concomitant dilute phase, in which dynamic material exchange takes place over short timescales [2, 21].

**Fig. 1:**
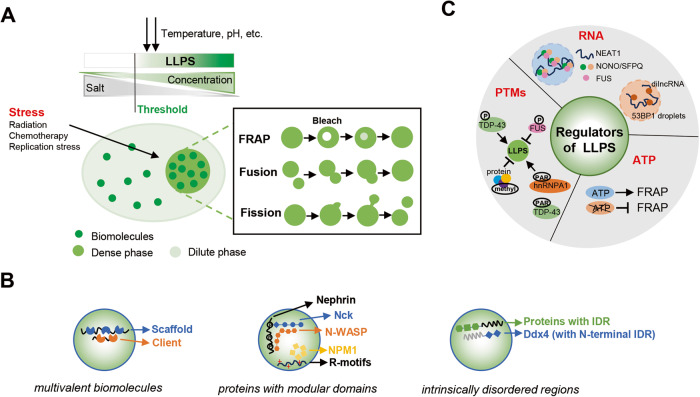
Principles of LLPS. **A** Biomolecular condensates exhibit liquid-like characteristics, including the capability of FRAP, fusion and fission events, which are influenced by concentration, salt, temperature, and pH. **B** LLPS of biomolecules are driven by multivalent binding interaction (left), modular domains (middle), and intrinsically disordered regions (right). **C** The regulators of LLPS include RNA, posttranslational modifications, and ATP.

Generally, LLPS has two distinct characteristics: dynamic diffusion and reversible compartmentalization. Based on this phenotype, fluorescence recovery after photobleaching (FRAP) is commonly considered a useful method to define a liquid-like biomolecular condensate in vivo. If the target protein labeled with a fluorescence tag exhibits spherical shapes and recovers from photobleaching, it is considered undergoing LLPS. Meanwhile, properties of droplet-like condensates such as fusion and fission help to distinguish liquid from solid or gel-like properties [22–24] (Fig. 1A).

Importantly, the occurrence of LLPS is highly dependent on the concentration and physicochemical identities of the biomolecules and the surrounding environment, such as temperature, pH, salt type, salt concentration, and the coexisting biological macromolecules. As usual, LLPS can only be triggered when the concentration of biomolecules reaches a threshold. Up to the threshold concentration, higher concentration of biomolecules, lower salt concentration in the physiological environment as well as suitable temperature and pH are more likely to promote phase separation [22, 25].

### Mechanisms of LLPS

Biological macromolecules such as proteins and nucleic acids are prone to undergo LLPS under certain conditions, many of which are not common in living cells. In fact, only a small percentage of macromolecules are capable of phase separation in physiological contexts. In recent years, using the advantages of super-resolution microscopy and electron microscopy, the common characteristics of biomolecules that undergo LLPS in living cells have gradually become known [26, 27] (Fig. 1B).

#### LLPS of multivalent biomolecules

Biomolecules that can phase separate under physiological conditions are usually multivalent and prone to make intra- or inter-molecular interactions [28, 29]. The interactions often occur between proteins or between proteins and nucleic acids. Such molecules containing multivalent proteins or nucleic acids are commonly referred to as scaffolds, which are indispensable for condensate formation. The clients are recruited to the scaffolds, bound to them, and separated into condensates. Clients themselves cannot undergo LLPS and are recruited and regulated by scaffolds. In addition, condensate formation is related to the stoichiometry and valency of scaffolds and clients [22, 30, 31].

#### LLPS of proteins with modular domains

Proteins composed of modular interaction domains such as Nephrin, Nck, and neural Wiskott-Aldrich Syndrome protein (N-WASP) undergo phase separation via the interactions between the modular domains. Nephrin comprises three phosphotyrosines (pTyrs) motifs that can interact with the SH2 domains in Nck. Also, Nck contains SH3 domains that can bind the proline-rich motifs in N-WASP. This Nephrin-Nck-N-WASP complex in the actin-regulatory signaling pathway can form phase separation-induced clusters on lipid bilayers upon LLPS [28, 32]. Nucleolar protein Nucleophosmin 1 (NPM1) contains an oligomerizing domain and negatively charged region, which enable NPM1 to assemble into polymers and bind to positively charged Arg-rich motifs containing proteins. Besides, NPM1 also interacts with multivalent nucleic acids and then undergoes LLPS [29, 33]. The number of the interaction modular domains and the affinity between them are important factors that promote LLPS. Generally, more interaction modular domains and higher affinity help to form larger condensates and facilitate LLPS at lower protein concentrations [28, 34].

#### LLPS of proteins with intrinsically disordered regions

Proteins with intrinsically disordered regions (IDRs) are usually prone to undergo LLPS. IDRs are devoid of constant tertiary structure, therefore is hard to exhibit abundant conformational changes and three-dimensional structures. However, IDRs often contain repeated elements, another type of weak multivalent interaction that mediate LLPS. The IDRs consist of low-complexity sequences and are composed of unvaried amino acids such as serine, glutamine, glycine, tyrosine, phenylalanine, and asparagine. Lacking diversity of amino acid types, IDRs often contain Gly/Ser-Phe/Tyr-Ser/Gly sequences and poly-Gln and poly-Asn regions, which may provide multiple motifs for interacting with RNAs and drive LLPS through dipolar interactions in cells [30, 35–38]. Emerging evidence shows that aromatic residues may play an important role in promoting LLPS. For example, the IDR in DDX4 can facilitate LLPS via cation-pi interactions between the Phe-Gly repeats and Arg residues. The N-terminal disordered tails of Ddx4 form condensates both in vivo and in vitro. Besides, many disordered proteins associated with membraneless organelles have the similar sequence characteristics as Ddx4 [39, 40]. In contrast, LLPS may be destroyed when aromatic residues mutate, such as the mutation of Tyr residues in BuGZ, FUS, and Nephrin intracellular domain [41–43]. Besides, interactions between the blocks of oppositely charged residues in IDR-containing proteins also contribute to the promotion of LLPS. In addition to the interactions among amino acids, IDR-containing proteins can also undergo LLPS via interactions involving the polypeptide backbone. For instance, RNA-binding proteins including FUS, hnRNPA2, and CIRBP undergo LLPS via interactions between stretches of b-strands occurring a few at a time [44–46].

### Regulators of LLPS

#### RNA

Increasing evidence suggests that RNAs play an essential regulatory role in triggering LLPS, such as mRNA, rRNA, and long non-coding RNA (lncRNA). RNAs usually serve as a bridge to connect multiple RNA-binding proteins (RBPs), which initiates LLPS by promoting the weak multivalent interaction between proteins or between RNAs and proteins [47, 48]. For example, lncRNA nuclear paraspeckle assembly transcript 1 (NEAT1) binds RBPs including non-POU domain containing octamer-binding protein (NONO) and splicing factor proline and glutamine (SFPQ), and promotes LLPS to form paraspeckles [49–51]. Damage-induced long non-coding RNAs (dilncRNAs) interact with 53BP1 and promote its LLPS at the DSBs sites [48]. Additionally, recent research suggests that multiple N^6^-methyladenosine (m^6^A) residues containing mRNAs may act as a multivalent scaffold to bind the YTHDF proteins, thereby driving its LLPS [52].

#### Posttranslational modifications

Posttranslational modifications (PTMs) are important regulators of protein function and stability, including phosphorylation, arginine methylation, acetylation, glycosylation, Poly(ADP-ribosyl)ation (PARylation), ubiquitylation and SUMOylation [53]. PTMs regulate phase separation by altering the multivalent interactions between macromolecules, recruiting other biomolecules into the condensate, or excluding other biomolecules from the condensate [54]. Research data indicate that protein arginine methylation at specific Arg residues results in the inhibition of condensate formation by diminishing multivalent cation-π interactions between Arg and aromatic residues. For example, arginine methyltransferase inhibitor enhances LLPS of FUS [55, 56]. In contrast to methylation, acetylation and phosphorylation can either promote or inhibit LLPS by affecting the protein conformation and protein-protein or/and protein-nucleic acid interactions. Phosphorylation of FUS inhibits its LLPS, while phosphorylation at Ser residues promotes TDP-43 LLPS [57–60]. In addition, PARylation is another PTM that is known to regulate the assembly and disassembly of RNA-binding proteins. Several lines of evidence reveal the important role of PARylation in regulating the LLPS of ribonucleoprotein (RNP) granules, such as TDP-43 and hnRNPA1 [61, 62].

#### ATP

Studies have shown that ATP affects the formation of LLPS by acting as an energy source or a hydrotrope that effectively changes the solubility of proteins. Many IDR-containing proteins that undergo LLPS exhibit FRAP, whereas depletion of ATP makes it difficult to recover from photobleaching. In line with this finding, several evidences reveal that the formation of nuclear condensate is significantly promoted in the presence of ATP. In contrast, a high concentration of ATP can also prevent LLPS of some proteins, e.g., Kang et al. reported that a high concentration of ATP may lead to the disassembly of FUS condensate [63–65].

### Functions of LLPS

As described above, biomolecular condensates are very common in cells, such as nucleolus, paraspeckles, and stress granules. These condensates provide independent spaces for a lot of biochemical reactions including enzymatic reactions, which are essential to cell survival. Accumulated studies have shown that LLPS plays important roles in regulating various cellular processes, such as DNA damage repair, gene expression, autophagy, chromatin organization, and biochemical pathway organization [21, 66, 67]. For example, Gibson et al. revealed that LLPS participated in establishing and maintaining chromatin compartments. The authors found that the reconstituted chromatin was able to form a dynamic liquid phase in the presence of physiologic salts, depending on the interactions between DNA and the histone H4. The linker DNA length, histone H1, and acetylation are the regulatory factors of chromatin LLPS. Their study suggested that LLPS plays an essential role in regulating the spatial organization of the genome [68].

Mounting studies have revealed that aberrant phase separation contributes to various human diseases such as tumorigenesis, neurodegenerative disorders, and some rare diseases. For instance, it has been reported that LLPS participates in neural development. The communications between the synapses are dependent on the molecular condensates via LLPS. Thus, LLPS plays a crucial role in signaling transmission in neuron [69]. Besides, microtubule-associated protein Tau can undergo LLPS. Pathologically, the sialylation of the Tau protein makes its condensation transit from liquid-like to solid-like that can’t be degraded, which further results in various neurodegenerative diseases such as Alzheimer’s disease [70]. Besides, LLPS is also involved in the cell tumorigenesis process and may be associated with cancers. Take the tumor suppressor speckle-type BTB/POZ protein (SPOP) for example, SPOP can form liquid-like droplets via interaction with its substrates, which can inhibit cancer-associated protein aggregation. In some tumors, SPOP often has oncogenic mutations that disrupt its ability to undergo LLPS, contributing to tumor progression [71]. Moreover, aberrant LLPS was also reported to be the driving force of rare diseases. Zhu et al. found that abnormal LLPS is related to Noonan syndrome, a rare disease induced by SHP2 mutations. Their research suggested that diseased-associated SHP2 mutants form puncta in cells via aberrant LLPS, accounting for MAPK hyperactivation [72].

## LLPS in DSBs repair

### A glance on DSBs repair mechanism

DSBs are the biggest threat to genome integrity. DSBs are mainly resulted from ionizing radiation (e.g., tumor radiotherapy), genotoxic chemicals (e.g., tumor chemotherapy), stalled replication forks, and reactive oxygen species (ROS). DSBs are repaired by either homologous recombination (HR) or nonhomologous end joining (NHEJ). HR is conducted in an error-free manner, that needs sister-chromatid or homologous chromosomes as a template and mainly functions in the S/G2 phase of cell cycle. In contrast, NHEJ ligates the DNA breaks directly in an error-prone way and works throughout the cell cycle. When DSBs occur, the MRE11/RAD50/NBS1 (MRN) complex binds to DSBs rapidly and functions as a DSBs sensor, which further recruits the protein kinase ataxia-telangiectasia mutated (ATM) and triggers ATM activation. ATM-mediated H2A.X phosphorylation at S139 serves as a platform for scaffold protein MDC1 that promotes the recruitment and retention of other DSBs repair factors, such as the MRN complex, RNF8, BRCA1, and 53BP1. For HR repair, the MRN complex catalyzes the generation of 3′ ssDNA that is further bound and protected by the RPA complex; consequently, the recombinase RAD51 replaces the RPA complex and forms RAD51/ssDNA filament, which is responsible for the search for homologous sequence and the invasion to dsDNA. For NHEJ repair, ku70/80 heterodimer binds to DSBs and recruits and activates protein kinase DNA-PK; further, the XRCC4/DNA LIG4/XLF complex is further recruited to complete the ligation of DSBs ends [12, 73–75].

### DSBs repair factors undergoing LLPS

#### 53BP1

53BP1 was originally identified as a p53 tumor suppressor binding protein [76, 77], although an increasing number of studies have focused on its roles in genomic integrity and DNA damage repair. 53BP1 is a large protein containing 1972 amino acids and several functional domains, including 28 Ser/Thr-Gln (S/T-Q) sites at its N-terminal, an oligomerization domain (OD), a Gly- and Arg-rich (GAR) motif, a tandem Tudor motif responsible for binding to dimethylated Lys20 of histone 4 (H4K20me2), a ubiquitylation-dependent recruitment (UDR) motif responsible for the interaction with ubiquitylated H2AK15 (H2AK15ub), and a BRCA1 carboxy-terminal (BRCT) repeats responsible for its interaction with DNA repair factors [78–80]. 53BP1 is a regulator of DSBs repair pathway choice, that promotes NHEJ and prevents HR [81, 82]. 53BP1 is reported to interact with key factors in different stages of DSB response, including DSBs sensor (MRN complex) [83], DSBs repair factors (BRCA1, RIF1) [84], DNA damage response transducers (ATM, ATR, CHK1/2) [83, 85–87], and cell cycle arrest-related factors (p53) [88, 89]. Various studies have shown that 53BP1 abnormal expression was correlated with carcinogenesis, thus targeting 53BP1 may be a promising strategy for treating cancer [90].

**Fig. 2:**
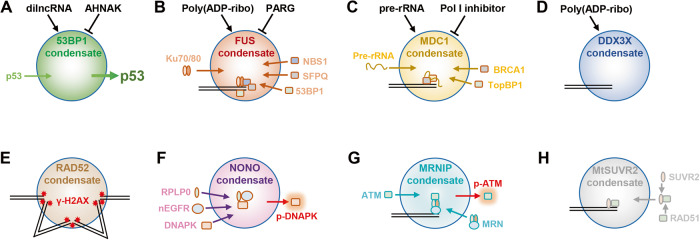
Functions and mechanisms of condensates in DSBs repair. **A** dilncRNAs induced 53BP1 condensates recruit and activate p53; AHNAK reduces the LLPS potency of 53BP1 by inhibiting 53BP1 oligomerization. **B** PAR accelerates FUS phase separation at DSBs site, which recruits Ku70/80, NBS1, SFPQ, 53BP1, and other repair factors; poly(ADP-ribose) glycohydrolase PARG is a suppressor of FUS condensation. **C** The interaction between MDC1 and pre-rRNA increases the multi-valency for MDC1 phase separation, resulting in MDC1 condensation at DSBs site and the recruitment of BRCA1, TopBP1; RNA Polymerase I inhibitor suppresses MDC1 condensation by diminishing pre-rRNA synthesis. **D** PAR-induced DDX3X condensation is essential for DDX3X accumulation at DSBs site. **E** Yeast RAD52 formed liquid-like condensates to cluster γ-H2A.X foci. **F** NONO condensates recruit ribosomal protein P0 (RPLP0), nuclear EGFR (nEGFR), and DNA-PK to enhance the DSB-induced activation and phosphorylation of DNA-PK. **G** MRNIP condensates recruit and concentrate MRN complex to DSB site to accelerate DSB sensing and end resection. **H** Histone methyltransferase SUVR2 in Medicago truncatula undergoes LLPS at DSBs site; it interacts with MtRAD51 and drives its phase separation, which enhances the stability of MtRAD51 proteins to facilitate error-free homologous recombination repair.

53BP1 is the first characterized phase-separated DSBs repair protein. Based on the CRISPR/Cas9-mediated endogenous 53BP1 tagging cells, Kilic et al. described the dynamic property and droplet-like behavior of 53BP1 condensates, presenting as frequent fusion and fission event. Also, the authors identified that the 53BP1 repair compartment at DNA lesions and DSBs sites exhibited the characteristics of liquid-liquid phase separation. As described above, the formation of 53BP1 condensates is influenced by physiological conditions, such as temperature, salt concentration, and osmotic pressure, while the upstream accumulation of γH2AX and MDC1 is not. Using optoDroplet, a tool of light-controlled LLPS, to perform gain-of-function experiments, the authors found that tumor repressor protein p53 was enriched in 53BP1 condensates. The BRCT domain of 53BP1 might participate in the assembly of p53 into optoDroplets. Moreover, the phase-separated 53BP1 compartments may contribute to the stabilization of p53 upon DNA damage. Disruption of 53BP1 condensates diminished the activation of p53-target genes, such as p21. Once the phase separation of 53BP1 was impaired, both DNA damage-induced p53 stabilization and p21 expression were decreased substantially [66]. Later, Pessina et al. also reported the LLPS property of 53BP1 condensates. In this study, they characterized damage-induced long non-coding RNAs (dilncRNA), a class of non-coding RNA synthesized at DSBs by a complete RNA polymerase II preinitiation complex, MED1, and CDK9, were required for the formation of DDR foci and 53BP1 condensates. The absence of dilncRNA is essential for stimulating LLPS of DDR factors (including 53BP1) in the shape of foci [48]. This study provides the mechanism of irradiation-induced 53BP1 phase separation at DSBs site. Further, scaffold protein AHNAK was identified as the inhibitor of 53BP1 phase separation [91]. AHNAK is a G1-phase-enriched interactor of 53BP1, that binds to the oligomerization domain of 53BP1 and diminishes its multimerization potential. Loss of AHNAK results in hyper-accumulation of 53BP1 on chromatin and enhanced phase separation, culminating in an elevated p53 response (Fig. 2A). Consistent with this report, Zhang et al. demonstrated that in normal cultured cells, 53BP1 condensates bound to heterochromatin and contributed to the maintenance of heterochromatin integrity and genome stability [92].

### Poly(ADP-ribose)

PARylation was first described more than 50 years ago. It is known as a DNA-dependent reaction that consumes nicotinamide adenine dinucleotide (NAD+) to build poly(ADP-ribose) (PAR) chains [93, 94]. The major enzyme catalyzing this reaction is PAR polymerase 1 (PARP1) [95], which regulates a number of biological processes including DNA repair [96, 97], carcinogenesis [98, 99], metabolism [100, 101] signaling [102, 103], gene transcription [104, 105] and cell death [105, 106]. In response to DNA damage, PARP1 quickly detects and binds to both single-strand breaks (SSBs) and DSBs and starts rapid PAR synthesis, which then recruits PAR-binding DDR factors for DNA damage repair [107, 108]. Additionally, there is accumulating evidence suggesting that many RNA-binding proteins (RBPs) are recruited to the sites of DSBs through interacting with PAR, leading to the recruitment of DSBs repair proteins [109–111].

It was recently proposed that functional interactions between PAR and RBPs are assembled through LLPS [109, 111, 112]. At the damaged DNA site, PAR serves as a scaffolding factor for the assembly of these RBPs that contain IDR, thereby initiating dynamic intracellular compartments for the spatial segregation of damaged DNA [113, 114]. This process relies on electrostatic interactions between positively charged arginine–glycine–glycine (RGG) repeats and negatively charged PAR, which is amplified by prone prion-like domains [112]. The assembly of RBPs at DNA break sites is dynamic and reversible mainly due to PAR glycohydrolase (PARG) activity [109]. PARG is sufficient to dissociate damaged DNA-rich compartments in vitro and initiates the nucleocytoplasmic shuttling of FUS in cells, providing a potential link between DNA repair and neurodegenerative diseases [109]. Therefore, local PAR formation can orchestrate intracellular phase transitions, allowing for their controlled formation, timely dissolution, and finally facilitating DNA repair.

### RNA-binding proteins

#### FUS

Fused in sarcoma (FUS) is an RNA-binding protein [115] that is involved in unique functions in gene transcription, RNA shearing, RNA transport, and translation [116]. In addition, FUS was also found to maintain genomic stability in response to DNA damage [117]. In particular, FUS is an early DDR factor, being recruited to the DSBs site dependent on PARP activity [118–120]. After being recruited, FUS directly interacts with DNA repair factors to facilitate DNA repair [121, 122]. Recent studies show that FUS undergoes LLPS, which is associated with the pathogenesis of the neurodegenerative disease [41, 56, 123]. An optogenetic tool, named as “optoDroplet”, has been used to study condensed phases driven by the IDRs of FUS. Lately, it was proposed that LLPS of FUS is necessary for the initiation of the DDR [124]. FUS is required for the recruitment of the DDR factors to DNA damage sites, including KU80, NBS1, 53BP1, SFPQ, and other RBP implicated in the DDR [125]. LLPS allows FUS to locally form droplet-like compartments, which further recruit these DDR factors and promote DSBs repair [126]. Moreover, the recruitment of FUS at the DSBs site is mediated by its interaction with PAR, which further promotes the assembly of damaged DNA-rich compartments (Fig. 2B) [109, 127].

#### NONO

The non-POU domain containing octamer-binding (NONO), an RNA and DNA binding protein, belongs to the Drosophila behavior human splicing (DBHS) family [128, 129]. NONO engages in various biological processes, including but not limited to DNA repair [130, 131], RNA splicing [132], RNA silencing [133], transcriptional regulation [128], and nuclear mRNA retention [134]. NONO is involved in DNA damage repair mainly through promoting the NHEJ pathway [135, 136]. NONO is also a PAR-binding protein and its recruitment to DNA damage sites is dependent on PARP activation [110]. Recently, NONO was found to bind to NEAT1 to form paraspeckle, a membraneless nuclear body driven by LLPS [49]. Our recent study reported that NONO condensates recruit nuclear EGFR and DNA-PK to enhance their interaction. Disruption of NONO droplets with LLPS inhibitor significantly reduced the DNA damage-induced EGFR/NONO as well as EGFR/DNA-PK complex, and suppressed the phosphorylation of DNA-PK at T2609 (Fig. 2F) [137].

#### DDX3X

DDX3X, a member of the DEAD-box RNA helicase family, plays a ubiquitous role in RNA metabolism [138, 139]. DDX3X also plays an important role in protecting genome integrity and its dysregulation is implicated in the development of cancer [140, 141]. Several studies have demonstrated that DDX3X is important for DNA repair [111, 142, 143]. Consistent with the observation of RNA-binding proteins recruited to DNA damage sites [144], DDX3X is recruited to the DSBs site in a PARP1-dependent manner. This recruitment is mediated by PAR-triggered LLPS of DDX3X. Inhibition of LLPS and CRISPR/Cas9-mediated knockout of PARP1 reduced DDX3X recruitment, suggesting a novel mechanism by which DDX3X regulates DSBs repair (Fig. 2D) [111].

### Other factors

#### Pre-rRNA

ncRNAs are essential regulators of DSBs repair factors condensation [145]. In addition to dilncRNAs that stimulate DSBs-induced 53BP1 condensation [48], pre-ribosomal RNA (pre-rRNA) also regulates the LLPS of DNA repair factors. Gan et al. find that pre-rRNA and ribosomal proteins, RNase MRP, and snoRNAs are localized in XY body and DSBs foci, and transient inhibition of RNA polymerase I abolishes foci formation of DSBs repair factor as well as DSBs repair. Mechanically, the FHA domain and PST repeats of MDC1 recognize pre-rRNA and mediate the formation of a phase-separated DSBs repair center (Fig. 2C) [146].

#### MRNIP

MRN-interacting protein (MRNIP) was first identified as a regulator of genome stability using an RNAi-based screen by Collis and colleagues [147]. It binds to the MRN complex and promotes MRN-mediated ATM activation and DNA end resection. Meanwhile, MRNIP was found to be a protector of replication fork [148]. Our recently published study reported that MRNIP formed liquid-like condensates in the nucleus, which recruited and concentrated the MRN complex [149]. When DSBs occur, MRNIP condensates move to the DSBs site and incorporate damaged DNA, enhancing the MRN complex-mediated ATM activation and DSBs end resection (Fig. 2G). The acceleration of MRNIP is correlated with the radioresistance of colorectal cancer. Consistent with our findings, Kazi et al. report that MRNIP has an essential function in spermatogenesis during meiosis I by forming drop-like accumulations interacting with the sex body [150].

#### Rad52

Yeast Rad52 protein is the functional analog of human BRCA2. After the generation of RPA-coupled ssDNA, Rad52 stimulates the removal of RPA and recruitment of Rad51 recombinase to ssDNA [151]. Rad51-coated ssDNA further searches and invades the homologous region of template DNA [152]. Oshidari et al. first show that in yeast, Rad52 protein undergoes LLPS at DSBs site. Rad52 proteins at different DNA damage sites assemble into liquid droplets and fuse into a repair center droplet via the action of petite DNA damage-induced intranuclear microtubule filaments (pti-DIMs). The resulting droplets tether the repair center to longer DIM-mediated mobilization of damaged DNA for repair [153]. Miné-Hattab et al. investigated the physical nature of Rad52 condensate using Single Particle Tracking (SPT) and Photo Activable Localization Microscopy (PALM) in *Saccharomyces cerevisiae* cells. They found that Rad52 molecules within condensate moved faster than the whole focus itself and the damaged DNA, indicating that most of Rad52 molecules within the DNA repair center were not bound to damaged DNA [154]. Their data further supports the phase-separated droplet properties of Rad52 at the DSBs site (Fig. 2E).

#### MtSUVR2

*Medicago truncatula* SUVR2 (MtSUVR2) is a plant-specific histone methyltransferase that catalyzes the conversion of histone H3 lysine 9 monomethylation (H3K9me1) to H3K9me2/3. The SET domain of AtSUVR2 in Arabidopsis has been shown to interact with histone H3, and AtSUVR4 also interacts with histone H3 [155, 156]. To investigate whether MtSUVR2 participates in DSBs repair, Liu et al. first confirmed that MtSUVR2-mediated H3K9me2 and heterochromatin formation affected DSBs repair. Further, they found that the DSBs-induced MtSUVR2 forming phase-separated condensate at the DNA damage site. The IDR1 and low-complexity domain of MtSUVR2 determined its LLPS, whereas its IDR2 domain determined the interaction with MtRAD51. Interestingly, MtSUVR2 condensates drive the LLPS of MtRAD51, which enhances the stability of MtRAD51 and facilitates HR-mediated DSBs repair (Fig. 2H) [157].

## Concluding remarks

Accurate and efficient DSBs repair is crucial for cell survival and genome integrity. For efficient repair, a large amount of repair factors is recruited and highly concentrated on DSBs end. However, the nucleases activated and required by DSBs repair are big threats to genome DNA, for which the DSBs repair process needs to be restricted in a compartmentalized space. The formation of phase-separated repair cores driven by LLPS supported this requirement. Up to now, LLPS has been shown to play important roles in different stages of DSBs repair, including DSBs sensing, repair initiation, and pathway choice.

The spatiotemporal regulation of repair factors recruitment and dissolving at DSBs end is required for DSBs repair. Our and other groups’ studies have found that many DSBs repair proteins undergo LLPS, which may form condensates at DSBs end and then recruit the downstream repair factors to the damaged site. However, do these LLPS factors interplay with each other? Is the physical-chemical interaction between different condensates required for the accurate spatiotemporal sequence of DSBs repair factors? For instance, Kakarougkas et al. showed that at the late stage of HR, 53BP1 condensates would be pushed out of the repair core by BRCA1 puncta, presenting as a phenomenon that 53BP1 condensates surrounding BRCA1 foci like a shell, but not just dissolved from DSBs [19]. Is this process an interplay between 53BP1 condensates and BRCA1 condensates? Additionally, Chiolo et al. reported that in the DSBs repair process of heterochromatic repetitive DNAs, heterochromatin undergoes dramatic expansion and dynamic protrusions to relocalize the HR repair center out of heterochromatin to associate with RAD51, thereby suppressing the excessive recombination of repeat sequences [158]. In the case that HP1α-mediated heterochromatin has been shown as a condensate driven by LLPS, is the relocalization an interplay between condensates of repair core and heterochromatin?

The survival benefit of PARP1 inhibitor in BRCA1-deficient breast cancer proved that the DNA repair pathway is a promising target for cancer treatment. Meanwhile, LLPS has been found to play pivotal roles in tumorigenesis [159, 160], and inhibition of global LLPS by 1,6-hexanediol treatment showed remarkable inhibition of tumor growth and induction of tumor cell death [161], indicating that LLPS may be a promising target for cancer treatment. While 1,6-hexanediol, a general LLPS inhibitor, is highly toxic to normal cells, the development of LLPS inhibitors with low toxicity and higher specificity is needed. However, IDRs, the key driving force of LLPS, lack a stable 3-dimensional structure. Although there are many web tools to analyze the disordered region of LLPS proteins, it is still a big challenge to develop drugs targeting LLPS. Recently, several small molecule inhibitors targeting IDRs have been reported [162, 163]. For instance, EPI analogs have been proven as androgen receptor (AR) IDR-specific binding inhibitors. The specificity and efficiency were verified by in vitro system, cellular experiments, and mouse model [164, 165]. Meanwhile, EPI-002, as a first-in-class drug that directly binds to an IDR, is the first IDR-targeted small compound being tested in the clinic (Clinical trial information: NCT02606123) [163].

Over the last ten years, studies on LLPS have risen as a hot field, accompanied by the emergence of new technologies for analyzing condensate properties, functions, and mechanisms. For condensate structure examination, we can use solution and solid-state nuclear magnetic resonance spectroscopy (NMR, ssNMR), small-angle x-ray scattering (SAXS), cryo-electron microscopy (cryo-EM), and atomic force microscopy (AFM), as well as single-molecule fluorescence microscopy (SMFM) techniques including fluorescence fluctuation spectroscopy (FFS) and single-molecule fluorescence resonance energy transfer (smFRET) [166]. For instance, using single-molecule microscopy in living cells, Miné-Hattab et al. characterized the different physical features of repair foci; most Rfa1 molecules are bound to the ssDNA while Rad52 molecules are free to explore the entire focus, reflecting the existence of a liquid Rad52 droplet around damaged DNA [154]. This method may be used to distinguish liquid-like droplets from foci. To identify the key domains of LLPS, many websites, e.g., PONDR, PLAAC, and PSPredictor, provide tools to predict the IDRs and the LLPS potency of target proteins [21, 22, 167]. In particular, OptoIDR provides a LLPS inducible tool to investigate LLPS function by gain-of-function [168]. However, there are still several unsolved points urging for new techniques. Firstly, loss-of-function experiments are mostly performed by deleting IDRs responsible for LLPS. However, as IDRs are usually relatively long polypeptides (from tens to hundreds of amino acids), removing them may disrupt protein structures and essential functional domains. Besides, we need a method to identify all LLPS proteins and evaluate their LLPS potency in cells, thereby stress-induced LLPS changes could be screened genome-wide.

Although LLPS has shed light on the mechanism underlying many biophysical features of condensates, some deep questions need to be further disclosed in future research. Firstly, the basic physical and chemical theory of LLPS remains largely unknown, which would be a key to developing the method of targeting LLPS in disease treatment. Additionally, in the bioengineering field, could the materials with inducible LLPS properties be used in biological research or clinical therapy? For example, vehicles incorporate drugs under the LLPS status while releasing the drugs under an inducible non-LLPS status in the tumor lesion to achieve a specific delivery of drugs.
